# IL‐13 determines specific IgE responses and SARS‐CoV‐2 immunity after mild COVID‐19 and novel mRNA vaccination

**DOI:** 10.1002/eji.202249951

**Published:** 2022-11-17

**Authors:** Stefan Meltendorf, Katrin Vogel, Christoph Thurm, Florian Prätsch, Annegret Reinhold, Jacqueline Färber, Hans‐Gert Heuft, Achim J. Kaasch, Thomas Hachenberg, Stefan Weinzierl, Burkhart Schraven, Dirk Reinhold, Monika C. Brunner‐Weinzierl, Holger Lingel

**Affiliations:** ^1^ Department of Experimental Pediatrics Otto‐von‐Guericke‐University Magdeburg Magdeburg Germany; ^2^ Institute of Molecular and Clinical Immunology Otto‐von‐Guericke‐University Magdeburg Magdeburg Germany; ^3^ Department of Anesthesiology and Intensive Care Medicine University Hospital Magdeburg Magdeburg Germany; ^4^ Institute of Medical Microbiology and Hospital Hygiene Otto‐von‐Guericke‐University Magdeburg Magdeburg Germany; ^5^ Department of Transfusion Medicine and Immunohematology University Hospital Magdeburg Magdeburg Germany; ^6^ Audio‐Communication Group Technical University Berlin Berlin Germany

**Keywords:** Allergy, COVID‐19, Cytokines, SARS‐CoV‐2, Vaccination

## Abstract

After recovery, mild and severe COVID‐19 diseases are associated with long‐term effects on the host immune system, such as prolonged T‐cell activation or accumulation of autoantibodies. In this study, we show that mild SARS‐CoV‐2 infections, but not SARS‐CoV‐2 spike mRNA vaccinations, cause durable atopic risk factors such as a systemic Th2‐ and Th17‐type environment as well as activation of B cells responsive of IgE against aeroallergens from house dust mite and mold. At an average of 100 days post mild SARS‐CoV‐2 infections, anti‐mold responses were associated with low IL‐13 levels and increased pro‐inflammatory IL‐6 titers. Acutely severely ill COVID‐19 patients instead showed no evidence of atopic reactions. Considering convalescents of mild COVID‐19 courses and mRNA‐vaccinated individuals together, IL‐13 was the predominant significantly upregulated factor, likely shaping SARS‐CoV‐2 immunity. Application of multiple regression analysis revealed that the IL‐13 levels of both groups were determined by the Th17‐type cytokines IL‐17A and IL‐22. Taken together, these results implicate a critical role for IL‐13 in the aftermath of SARS‐CoV‐2 mild infections and mRNA vaccinations, conferring protection against airway directed, atopic side reactions that occur in mildly experienced COVID‐19.

## Graphical abstract

To investigate atopic immune reactions in response to SARS‐CoV‐2 mRNA vaccinations or mild infections serum IgE and cytokines were analyzed. An atopic environment prone to reactions against aeroallergens was revealed in convalescents but not vaccinated. IL‐13 turned out to be critical to confer SARS‐CoV‐2 immunity and protection to these reactions.

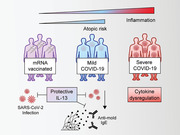

## Introduction

Controlling the pandemic COVID‐19 outbreak has put tremendous efforts in deciphering anti‐SARS‐CoV‐2 immune responses and in establishing immunity by novel mRNA vaccination. As a hallmark, SARS‐CoV‐2 infections can elicit profound and lasting cytokine responses, in which Th2 responses pathologically contribute to inflammation and lung damage in severe COVID‐19 patients [1]. Moreover, convalescents that resolved mild SARS‐CoV‐2 infections show an altered immune composition, in which the Th2‐associated cytokine IL‐33 has been reported to persist in serum beside elevated levels of circulating autoantibodies [2, 3]. The induction and the role of Th2 responses in mild SARS‐CoV‐2 infections still remain elusive, especially whether Th2‐type immunity could promote unfavorable IgE antibody responses against common aeroallergens [4, 5]. For instance, bioinformatics approaches revealed putative cross‐reactive allergen‐derived epitopes, such as from *Aspergillus fumigatus*, in the SARS‐CoV‐2 proteome [6]. To protect individuals against severe COVID‐19, mRNA vaccination has been established as an efficacious tool [7]. However, due to novelty of this approach and the consequent lack of data, concerns regarding lasting side effects such as myocarditis or induction of allergy might be raised [8, 9]. Therefore, we assessed systemic cytokine and antibody abundancies to investigate the induction of Th2 and IgE antibody responses, especially against non‐seasonal common allergens in acute severe, convalescent mild COVID‐19 patients, and mRNA‐vaccinated individuals.

## Results and Discussion

### Th2 and Th17 biased immune composition after resolving of mild SARS‐CoV‐2 infections

**Figure 1:**
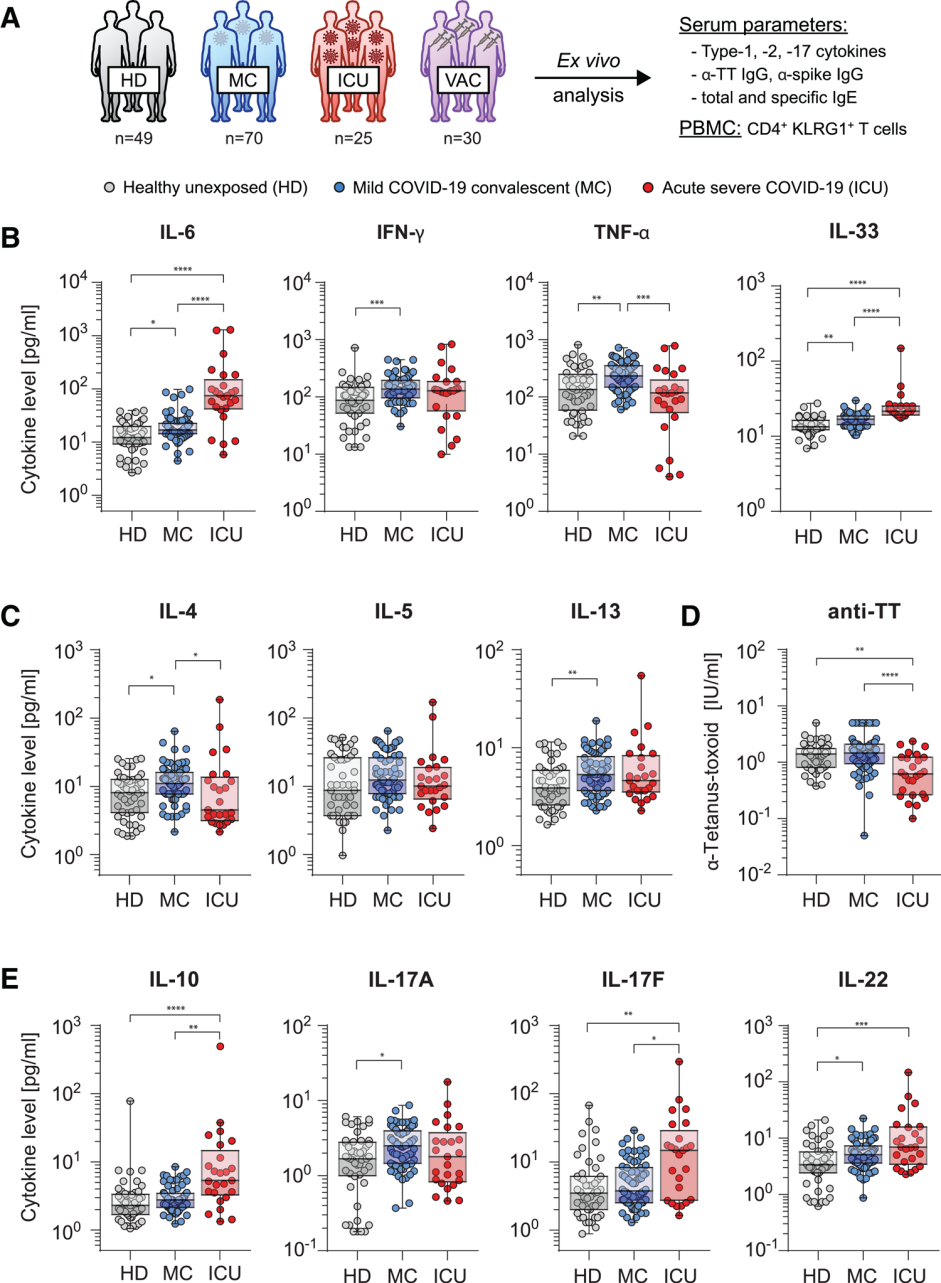
Defined signature of elevated systemic cytokine levels in convalescents of mildly experienced SARS‐CoV‐2 infections. (A) Overview of experimental design on the study cohorts of healthy unexposed donors (light gray, HD), mild COVID‐19 convalescents (blue, MC), acute severe COVID‐19 patients (red, ICU), and SARS‐CoV‐2 spike mRNA‐vaccinated donors (VAC, violet). (B,C,E) Serum cytokine levels of healthy unexposed donors (light gray, HD), mild COVID‐19 convalescents (blue, MC), and acute severe COVID‐19 patients (red, ICU) quantified by flow cytometric multiplex bead‐based assay. (D) Anti‐Tetanus‐toxiod IgG titers of HD, MC, and ICU. Data points represent donors with median, interquartile, and range. *p < 0.05; **p < 0.01; ***p < 0.001; ****p <0.0001; *p*‐values were calculated using Kruskal‐Wallis test with Dunn's post hoc test.

This study comprised 49 healthy unexposed donors (HD) that neither showed symptoms nor detectable anti‐SARS‐CoV‐2 antibodies, 70 individuals who recovered from mild SARS‐CoV‐2 infections (MC, venipuncture at mean day 100 post infection) and 25 acute severe COVID‐19 patients treated at intensive care unit (ICU, venipuncture at mean day 13 post infection) as well as 30 uninfected donors that were vaccinated twice with full‐length spike protein‐encoding mRNA vaccines (VAC, venipuncture at day 28 after second vaccination) (Fig. 1A, Table S1). To assess the long‐term impact of mild SARS‐CoV‐2 infections on the immune system of convalescents (MC) we found the pro‐inflammatory biomarkers IL‐6 and TNF‐α as well as Th1 cytokine IFN‐γ, but not IL‐2 levels elevated in comparison to HD (Fig. 1B, data not shown) [10]. In both MC and ICU elevated IL‐33 levels show an association to lung injury [2] and could promote Th2 and Th17 differentiation (Fig. 1B right) [11, 12]. IL‐33 could then impinge on type‐2 immunity mediating lung‐resident ILCs to restore airway epithelial integrity [13]. To analyze the impact of SARS‐CoV‐2 infections especially in mildly experienced convalescents we assessed the Th2‐associated cytokines and detected increased IL‐4 and IL‐13, but not IL‐5 levels in MC (Fig. 1C). Considering the elevated Th1 response in MC, these cytokines could be induced via bystander activation as seen in other viral infections [14, 15]. To investigate bystander activation that promotes antibody production such as against vaccine antigens, we determined anti‐Tetanus‐toxoid titers with no difference between MC and HD but a significant reduction in ICU, which could be due to lymphopenia in the latter (Fig. 1D) [16]. Further, the ICU showed elevated IL‐10 levels that could play a role in the disease severity in these patients (Fig. 1E) [17]. Together, these data point toward a specifically induced long‐lasting Th2 environment in MC resulting from Th2 memory progenitors that could set the basis for atopic immune reactions such as sensitization against commonly inhaled allergens in the respiratory tract as the primary site of SARS‐CoV‐2 infections [18, 19].

In this regard, Th17 cytokines play a central role in lung inflammation and can support allergy induction [20, 21]. Therefore, we expanded our analysis on Th17‐associated cytokines. Ultimately, significantly elevated levels of IL‐17A were detected in MC whereas ICU showed higher levels of IL‐17F, when compared to those of HD (Fig. 1E). These data indicate that, in line with the literature, IL‐17A supports the Th2 cytokine expression in MC whereas IL‐17F could dampen the Th2 response, however leads to neutrophil recruitment and activation in ICU [22]. In line with long lasting cytokine responses even after resolving of SARS‐CoV‐2 infection, increased IL‐22 levels in MC and ICU indicate ongoing processes in order to restore lung epithelia integrity (Fig. 1E right) [23].

### Elevated IgE antibodies against common aeroallergens in mild COVID‐19 convalescents

Taking into account that binding of IL‐4 to IL‐4Rα occurs with high affinity, meaning that very low concentrations of IL‐4 can maximally occupy the receptor chains at a given cell surface, the increased IL‐4 levels in MC could result in profound effects on the immune response [4]. As a result of the elevated IL‐4 and IL‐17A cytokine levels of MC, these individuals are prone to sensitization to commonly inhaled aeroantigens [18, 24, 25]. Therefore, we assessed serum levels of IgE against non‐seasonal allergens from house dust mite (HDM) and mold.

**Figure 2:**
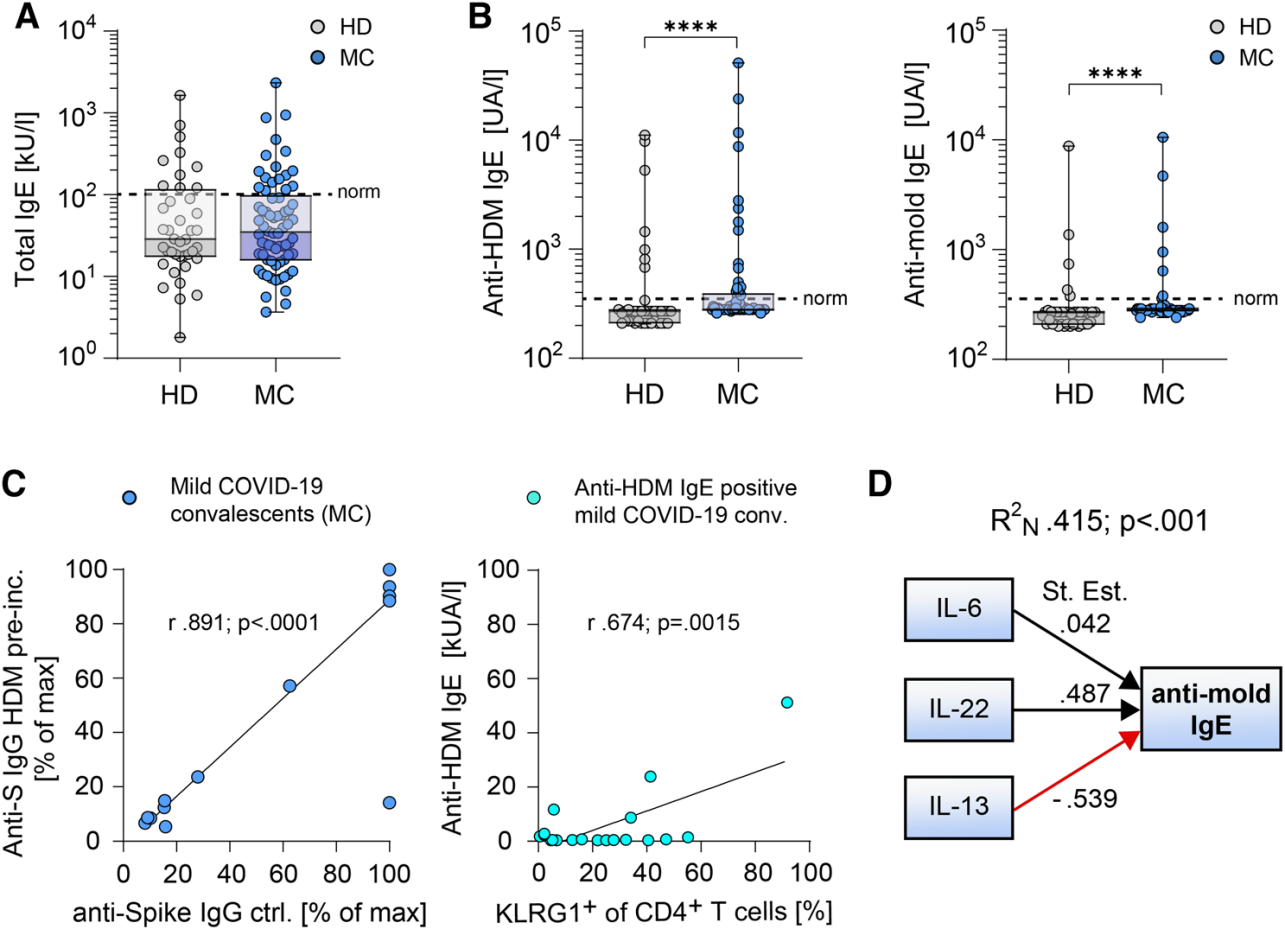
Specifically elevated IgE antibody levels in mild COVID‐19 convalescents. (A,B) Total IgE (A) and anti‐HDM (B *left*) or anti‐mold (B *right*) antibody levels of healthy unexposed donors (light gray, HD) and mild COVID‐19 convalescents (blue, MC). (C) Binding capacity of anti‐Spike IgG antibodies of MC sera pre‐incubated with HDM peptides (*left*). Simple linear regression analysis of positive anti‐HDM IgE antibody levels to frequencies of KLRG1^+^ CD4^+^ T cells of MC (*right*). (D) Path diagram of logistic regression of cytokines regulating anti‐mold IgE antibodies in MC. Data points represent donors with median, interquartile, and range. Numbers indicate *r*, adjusted *R* ^2^, stand. Estimate, or *p*‐values, respectively. ****p <0.0001; *p*‐values were calculated using two‐tailed Mann–Whitney test (A and B) or regression analysis (C and D).

Despite no detectable overall changes in total IgE antibody levels (Fig. 2A), specific IgE antibody levels against HDM or mold where significantly increased in MC when compared to HD (Fig. 2B). Of note, the binding capacity of anti‐SARS‐CoV‐2 spike IgG antibodies was not impaired by pre‐incubation with HDM antigens, excluding cross‐reactivity between spike and HDM antigens (Fig. 2C left). Intriguingly, a correlation between anti‐HDM IgE levels and expression of the exhaustion marker KLRG1 on CD4^+^ T cells could be detected in anti‐HDM IgE positive MC (Fig. 2C right), implicating that elevated specific IgE levels in the context of exhausted T‐helper cell responses represent a unique characteristic specificity to the recovery from SARS‐CoV‐2 infections [5, 26]. To identify factors that determine the risk to develop specific IgE responses in MC, logistic regression analysis revealed that anti‐mold IgE levels are significantly promoted (R^2^_N_ = 0.415; p<0.001) by IL‐6 and IL‐22, but prevented by IL‐13 indicating a protective role for IL‐13 in this regard (Fig. 2D). Of note, MC with known established allergic airway diseases did not preferentially show high IL‐13 levels (p = 0.259) (data not shown).

### No atopic risk in acute severe COVID‐19 patients and mRNA vaccinated donors

**Figure 3:**
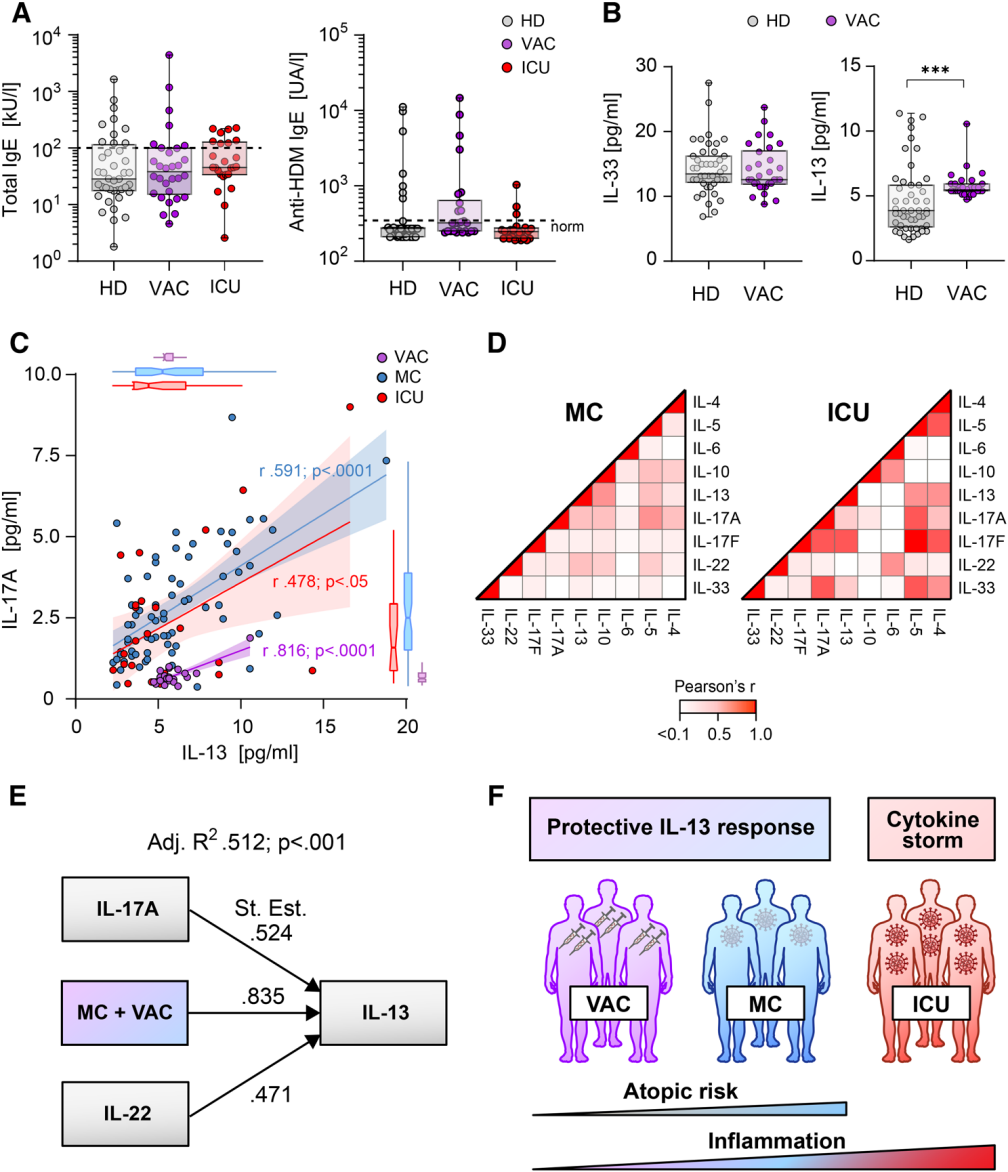
IL‐17A and IL‐22 equally drive protective IL‐13 in MC and VAC. (A) Total IgE (*left*) and anti‐HDM (*right*) antibody levels of HD (light gray), SARS‐CoV‐2 spike mRNA‐vaccinated donors (VAC, violet), and ICU (red). (B) Cytokine levels in sera of HD and VAC quantified by flow cytometric multiplex bead‐based assay. (C) Correlation of serum IL‐13 and IL‐17A levels in VAC, MC, and ICU. (D) Correlation heat map of serum cytokines of MC and ICU. White and red represent low and high correlation coefficients, respectively. (E) Path diagram of multiple linear regression analysis of factors regulating IL‐13 in both MC and VAC. (F) Illustration for atopic risk and inflammation induced by SARS‐CoV‐2 infection or vaccination. Data points represent donors with median, interquartile, and range. Numbers indicate *r*, adjusted *R* ^2^, stand. Estimate, or *p*‐values, respectively. ***p < 0.001; *p*‐values were calculated using Kruskal–Wallis test with Dunn's post hoc test (A), two‐tailed Mann–Whitney test (B), or regression analysis (C‐E).

Despite elevated IL‐4 levels in ICU patients, neither their total IgE nor their anti‐HDM IgE levels differed from those of HD (Fig. 3A). Considering that ICU patients show severe T‐cell lymphopenia, the lack of atopic events might be in part explained by absence of sufficient numbers of Th2 cells [16]. To protect against severe SARS‐CoV‐2 infections, mRNA vaccines proofed as efficacious; however, administration of vaccine components like polyethylene glycol raised concerns of possible allergic side reactions [8]. Therefore we similarly assessed serum parameters in individuals that had been vaccinated twice with SARS‐CoV‐2 spike mRNA (VAC; n = 30). Analyzing IgE antibodies four weeks after the second vaccination, no increased levels of total IgE or anti‐HDM IgE antibodies were detectable (Fig. 3A). In line, vaccinated donors did not show any signs of inflammation, as the cytokine levels of IL‐6, IL‐4, IL‐5, IL‐10, IL‐17, IL‐22, and IL‐33 remained similar to those of the healthy unvaccinated group (Fig. 3B left, data not shown). However, despite low levels of inflammation a marked increase in IL‐13 abundance was detectable in this group (Fig. 3B right). In line with a reported role for IL‐13 as a specific recall marker [27], these findings affirm the expression of IL‐13 as a characteristic feature of SARS‐CoV‐2 spike mRNA vaccination. Of note, the MODERNA mRNA vaccinated individuals showed higher IL‐13 levels (p = 0.033) compared to those donors vaccinated with BioNTech/Pfizer mRNA vaccine; however, there were no differences in anti‐HDM or IL‐33 levels between these subgroups (data not shown).

### Role of IL‐13 in immunity to SARS‐CoV‐2 infections

To investigate the overall function of specific cytokine composition after mild and severe SARS‐CoV‐2 infections or mRNA vaccination we performed regression analysis of the assessed parameters. To identify common factors that are involved in the immunity to SARS‐CoV‐2 infections we focused on IL‐13 as its levels are compared to HD particularly elevated in presumably protected MC and VAC, but not in imperiled ICU. Given this prominent role of IL‐13, we sought to characterize the parameters that provoke high levels of this cytokine. As IL‐17A could induce IL‐13, a highly significant correlation between those two factors (r>0.5; p<0.001, respectively) could be observed in MC and VAC but not in ICU (Fig. 3C) [28]. Instead, the acute severely infected individuals showed the signs of a cytokine storm characterized by other multiple strong correlations in‐between when compared to MC (Fig. 3D). However, IL‐17A levels alone were not sufficient to explain IL‐13 expression when both MC and VAC were considered together. Multiple correlation analysis revealed (adjusted R^2^ = 0.512; p<0.001) that IL‐17A and IL‐22 were highly significant and equally important factors in the expression of IL‐13 in both study groups that are assumed to possess protective anti‐SARS‐CoV‐2 immunity (Fig. 3E). Taken together, these data highlight type‐17‐driven IL‐13 as a characteristic feature of MC and VAC immunity to mild SARS‐CoV‐2 infections.

## Concluding remarks

In summary, our data show that there exist unique interrelated systemic cytokine sets of defined Type‐2 and Type‐17‐responses that persist in convalescents even 100 days post mildly experienced SARS‐CoV‐2 infections (MC) with signatures that are distinct from those of vaccinated or acute severe ICU patients, respectively. In particular, mild SARS‐CoV‐2 infections could set the basis for atopic immune events such as IgE production against common aeroallergens that were absent under strong inflammatory conditions in acute severely infected COVID‐19 patients (Fig. 3F). Especially elevated IL‐4 titers, effective even at low concentrations, may promote atopic sensitization after infection [4], that could be thwarted by IL‐13 expression (Fig. 2D).

Additionally, the Th2‐type cytokine IL‐13 appeared as a commonly regulated factor in MC and VAC that both are assumed to possess immunity against SARS‐CoV‐2 infections (Fig. 3F). This particular role of IL‐13 is supported by its recently described function in SARS‐CoV‐2 specific recall responses after infection and vaccination [27]. As IL‐13 is associated with fast viral clearance [29] and patients with asthma driven by IL‐13 have a lower risk of death by COVID‐19 [30], the presence of a persistent concentration of IL‐13 in vaccinated or convalescent individuals may confer an advantage in the case of exposure to SARS‐CoV‐2, e.g. by down‐regulating ACE2 expression and attenuation of viral entry, replication, and spreading [31, 32, 33]. Thus, our data suggest that IL‐13 levels in sera should be included in the assessment of protective immunity due to resolved infection or vaccination.

## Material and methods

### Study cohorts and experimental design

The Ethics Board of the University of Magdeburg approved this study (certificates 159/18, 67/21). All patients, healthy donors or relatives of severe COVID‐19 patients provided written informed consent in accordance with the declaration of Helsinki. This study enrolled 70 mild COVID‐19 convalescents, 25 acute COVID‐19 patients at ICU, 49 presumed unexposed volunteers, and 30 uninfected donors that were vaccinated twice with either full‐length spike protein‐encoding mRNA vaccines from MODERNA (mRNA‐1273) (n = 22) or BioNTech/Pfizer (BNT162b2) (n = 8) (Table S1). Venipunctures of SARS‐CoV‐2 infected or unexposed individuals as well as vaccinated donors were conducted between April 2020 to May 2021. The venipunctures were performed in convalescents of mildly experienced SARS‐CoV‐2 infections (MC) at mean day 100 ± 38 days after positive test, in severe acutely infected patients (ICU) at mean day 13 ± 5 days after positive test, and in mRNA‐vaccinated donors at 28 days after the second vaccination. Different study group sizes were processed, as healthy unexposed individuals (HD) were only recruited within a limited time window with less than 10 infected individuals per 100,000 inhabitants. All donors were tested for the presence of SARS‐CoV‐2 RNA and/or anti‐SARS‐CoV‐2 spike or nucleocapsid IgG. HD had neither symptoms nor detectable antibodies against spike or nucleocapsid protein. Donors with a history of HDM allergy were excluded for anti‐HDM IgE analysis.

### Sample preparation and analysis

Serum separation was performed from whole blood samples by centrifugation of serum separation tubes (BD). Peripheral blood mononuclear cells (PBMC) were isolated by density‐gradient sedimentation using Pancoll (PAN Biotech) as previously described [3]. PBMC *ex vivo* analysis by flow cytometry was performed on a FACS Canto II with FACSDiva (BD Biosciences) and Flowjo software (Flowjo) by using specifically labeled antibodies against following molecules (clone names in parentheses): CD4 (RPA‐T4), CD3 (SK7), and KLRG1 (1D11) (all Biolegend).

Serum cytokine levels were assessed by LEGENDplex fluorescent bead‐based assay (Biolegend) according to the manufacturer's instructions (Suppl. Fig. 1A). Measurements were performed on a FACS Canto II (BD Biosciences) and quantified by LEGENDplex Data Analysis Software (Biolegend). Quantification and detection limits were calculated on standard curves (Suppl. Fig. 1B); detection limit for each cytokine: *IL‐4*: 0.82 pg/ml, *IL‐5*: 1.17 pg/ml, *IL‐6*: 1.95 pg/ml, *IL‐10*: 0.36 pg/ml, *IL‐13*: 0.75 pg/ml, *IL‐33*: 7.76 pg/ml, *IFN‐γ*: 10.47 pg/ml, *TNF‐α*: 12.25 pg/ml, *IL‐17A*: 0.29 pg/ml, *IL‐17F*: 0.89 pg/ml, and *IL‐22*: 0.47 pg/ml.

The quantitative detection of α‐Tetanus‐toxoid IgG in serum samples was performed in an accredited procedure by the Institute of Medical Microbiology and Hospital Hygiene (Otto‐von‐Guericke‐University Magdeburg) with SERION Tetanus IgG ELISA on an Immunomat (Virion∖Serion). The measurements of total and allergen specific IgE against house dust mix (contains a mix of Hollister‐Stier Labs., *Dermatophagoides pteronyssinus*, *Dermatophagoides farinae*, and *Blatella germanica*) and mold mix (contains a mix of *Penicillium chrysogenum*, *Cladosporium herbarum*, *Aspergillus fumigatus*, *Candida albicans*, *Alternaria alternata* and *Setomelanomma rostrate*) (Thermo Fisher Diagnostics) in serum samples were conducted using the ImmunoCAP system (Phadia). For SARS‐CoV‐2 cross reactivity tests the serum samples where pre‐incubated with 10 μg/ml HDM peptides (Citeq biologics) and binding to spike protein was then determined with the LEGEND MAX ELISA Kit (Biolegend) according to the manufacturer's instructions.

### Statistical analysis

Statistical analyses were performed using Prism9 (GraphPad) or R with Jamovi v. 2.2.2. Outliers were excluded by Grubb's test. In the case of values below the sensitivity of the test, these data points were calculated on the mean between 0 and the detection limit. Normal distribution of data sets was tested with Shapiro–Wilk test and significance analysis were accordingly performed using two‐tailed Mann–Whitney *U*‐test or using Kruskal‐Wallis test with Dunn's correction for multiple comparisons. The interrelation between variables was analyzed by simple (Fig. 2, 3) or multiple linear regression controlled by ordinal regression analysis (Fig. 3E), or logistic regression analysis (Fig. 2D).

## Author contributions

H.L., S.M., and M.C.B.‐W. designed research; H.L., S.M., K.V., C.T., and J.F. performed research; H.L., S.M., S.W., and M.C.B.‐W. analyzed data; A.R., F.P., T.H., H.‐G.H., and A.J.K. contributed new reagents/analytic tools; H.L., S.M., and M.C.B.‐W. wrote the paper; S.W., K.V., D.R., and B.S. edited the manuscript.

## Conflict of interest

The authors declare no commercial or financial conflicts of interest.

### Ethics approval

This study was approved by the ethics committee of the Otto‐von‐Guericke‐University of Magdeburg (certificates 159/18, 67/21).

### Patient consent statement

All participants have provided written informed consent.

### Peer review

The peer review history for this article is available at https://publons.com/publon/10.1002/eji.202249951

**Abbreviations**
COVID‐19: coronavirus disease 2019
HDM: house dust mite
ICU: intensive care unit
IgE: immunoglobulin E
SARS‐CoV‐2: severe acute respiratory syndrome coronavirus

## Supporting information

Supporting Information

## Data Availability

The data that support the findings of this study are available on request from the corresponding author. The data are not publicly available due to privacy or ethical restrictions.
