# Supplementary material for: IL‐13 determines specific IgE responses and SARS‐CoV‐2 immunity after mild COVID‐19 and novel mRNA vaccination
Source: Eur J Immunol. 2022 Nov 17;52(12):1972–9. doi: 10.1002/eji.202249951 (PMC9874813; doi:10.1002/eji.202249951)
Supplement: Supplementary file 1 — Supporting Information [file EJI-52-1972-s001.pdf]

Supplementary TABLE 1. Clinical characteristics of the cohorts

Characteristics	Unexposed (n=49)	Mild COVID-19 (n=70)	Severe (ICU) COVID-19 (n=25)	mRNA Vaccinated (n=30)	Reference range
Timepoint of venipuncture – days after pos. test / 2nd vac.	-	100 ± 38	13 ± 5	28	
Age – y. (range)	46±13 (17-79)	48±12 (15-71)	68±10 (59-85)	38±13 (22-65)	
Gender – no. (%)					
Male	16 (32.6)	29 (41.4)	15 (60)	12 (40)	
Female	33 (77.4)	41 (58.6)	10 (40)	18 (60)	
Smoker – no. (%)			N/A	N/A	
Non smoker	36 (73.5)	46 (65.7)			
Smoker	9 (18.4)	13 (18.6)			
Former Smoker	4 (8.1)	11 (15.7)			
Personal history of autoimmune disease – no. (%)	2 (4)	5 (7.1)	4 (16)	0 (0)	
Co-morbidities such as – no. (%)				N/A	
Hypertension	11 (22.4)	13 (18.6)	16 (64)		
Diabetes	0 (0)	4 (5.7)	5 (20)		
Allergic airway dis.	13 (26.5)	19 (27.1)	1 (4)	16 (53.3)	
COVID-19 Severity Scale – no. (%)					
Asymptomatic		9 (12.9)	-		
Mild		61 (87.1)	-		
Moderate		-	-		
Severe / critically ill at ICU		-	25 (100)		
Mild COVID-19 Symptoms – no. (%)					
Fever		34 (48.6)			
Cough		49 (70)			
Shortness of breath		27 (38.6)			
Loss of smell and taste		48 (68.6)			
Antibody Test positives – no. (%)					
IgG – S	0 (0)	51 (72.8)	23 (92)	30 (100)	< 7 U/ml
IgE total	10 (21)	17 (24.3)	8 (32)	6 (20)	< 100 kU/l
IgE – housedust	7 (14.3)	21 (30)	2 (8)	13 (43.3)	< 350 UA/l
IgE – mold	5 (10)	8 (11.4)	1 (4)	6 (20)	< 350 UA/l

SUPPLEMENTARY FIGURE

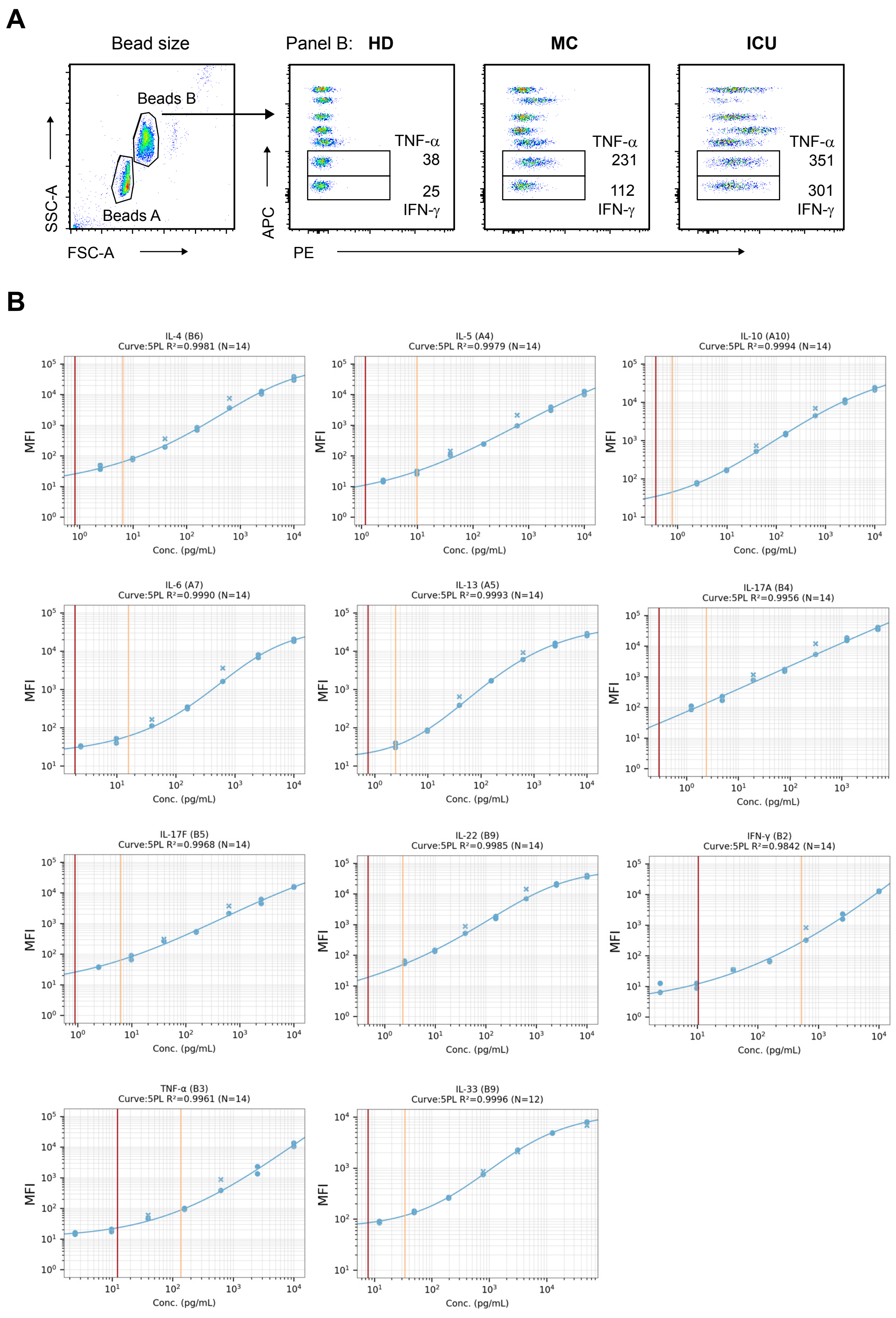

Suppl. Figure 1 (A) Representative dot plots of LEGENDplex serum cytokine analysis of healthy unexposed donors (HD), convalescent mild (MC), and acute severe COVID-19 patients (ICU). Analysis is shown for the Th Cytokine Panel cytokines TNF-α and IFN-γ among other cytokines. Each population represents the acquisition of a certain cytokine, numbers represent the mean fluorescence intensity (MFI), respectively. (B) Standard curve concentration range measured for each cytokine in the LEGENDplex assay, respectively.
